# Supplementary material for: Effect of the Protein Corona Formation on Antibody Functionalized Liquid Lipid Nanocarriers
Source: Int J Mol Sci. 2023 Nov 25;24(23):16759. doi: 10.3390/ijms242316759 (PMC10706289; doi:10.3390/ijms242316759)
Supplement: Supplementary file 1 [file ijms-24-16759-s001.zip › ijms-2733040-SI.pdf]

## Supporting Information

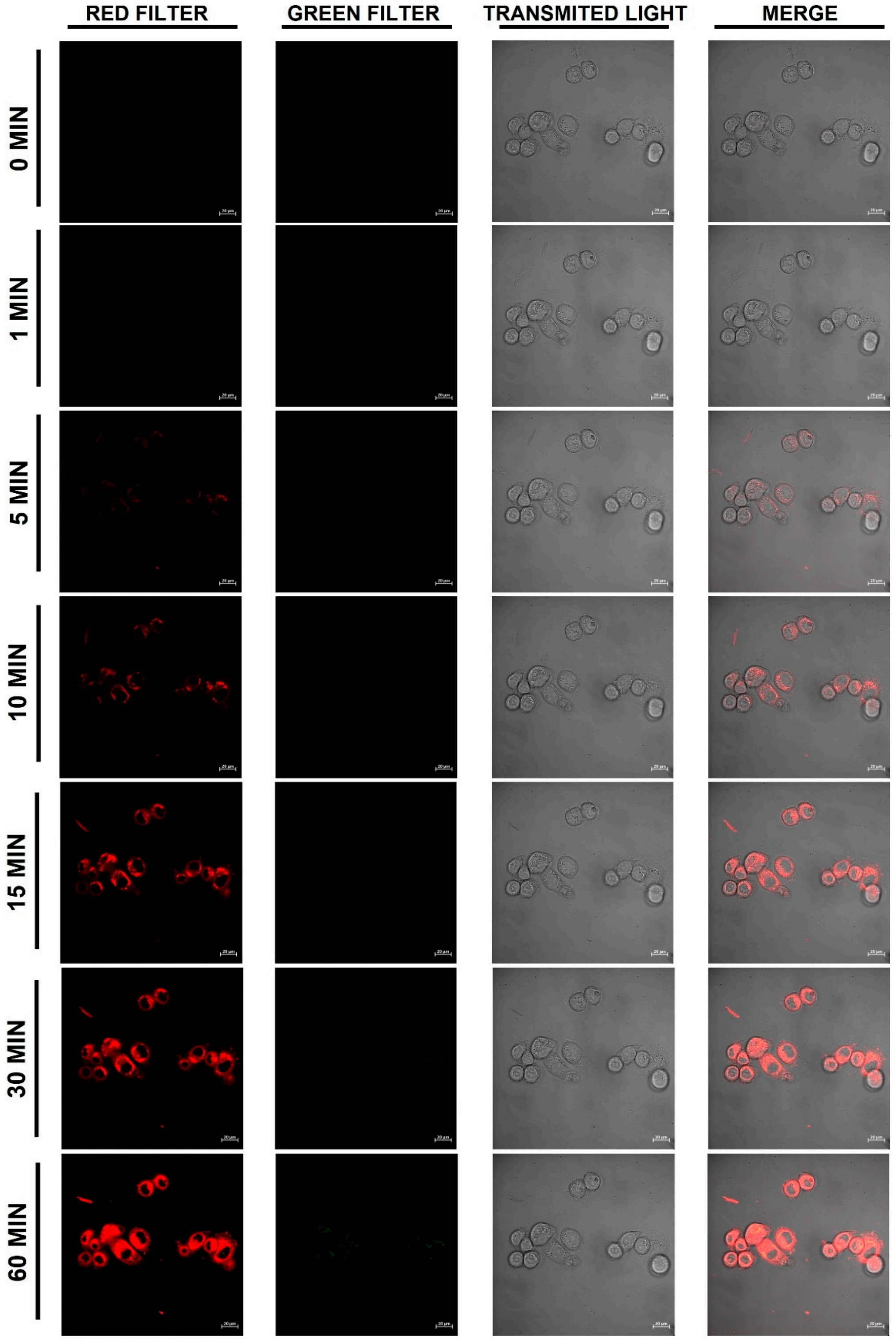


Figure S1. *Confocal microscopy of SKBR3 (scale bar = 20 μm) incubated with NR-LLNCs. Bare LLNCs were used as control.*

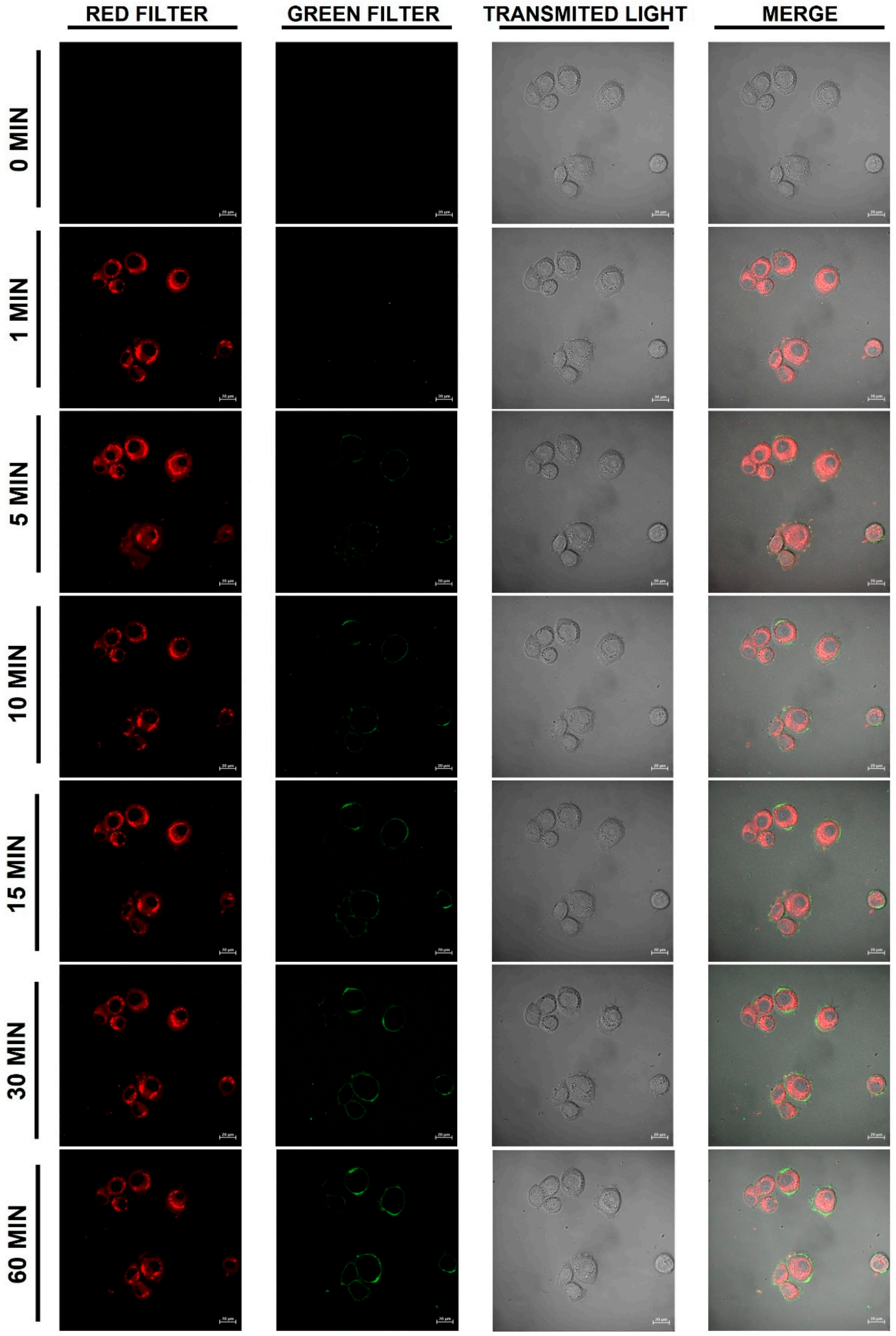


Figure S2. *Confocal microscopy of SKBR3 (scale bar = 20 µm) incubated with NR-LLNCs-HER2. Bare LLNCs were used as control.*

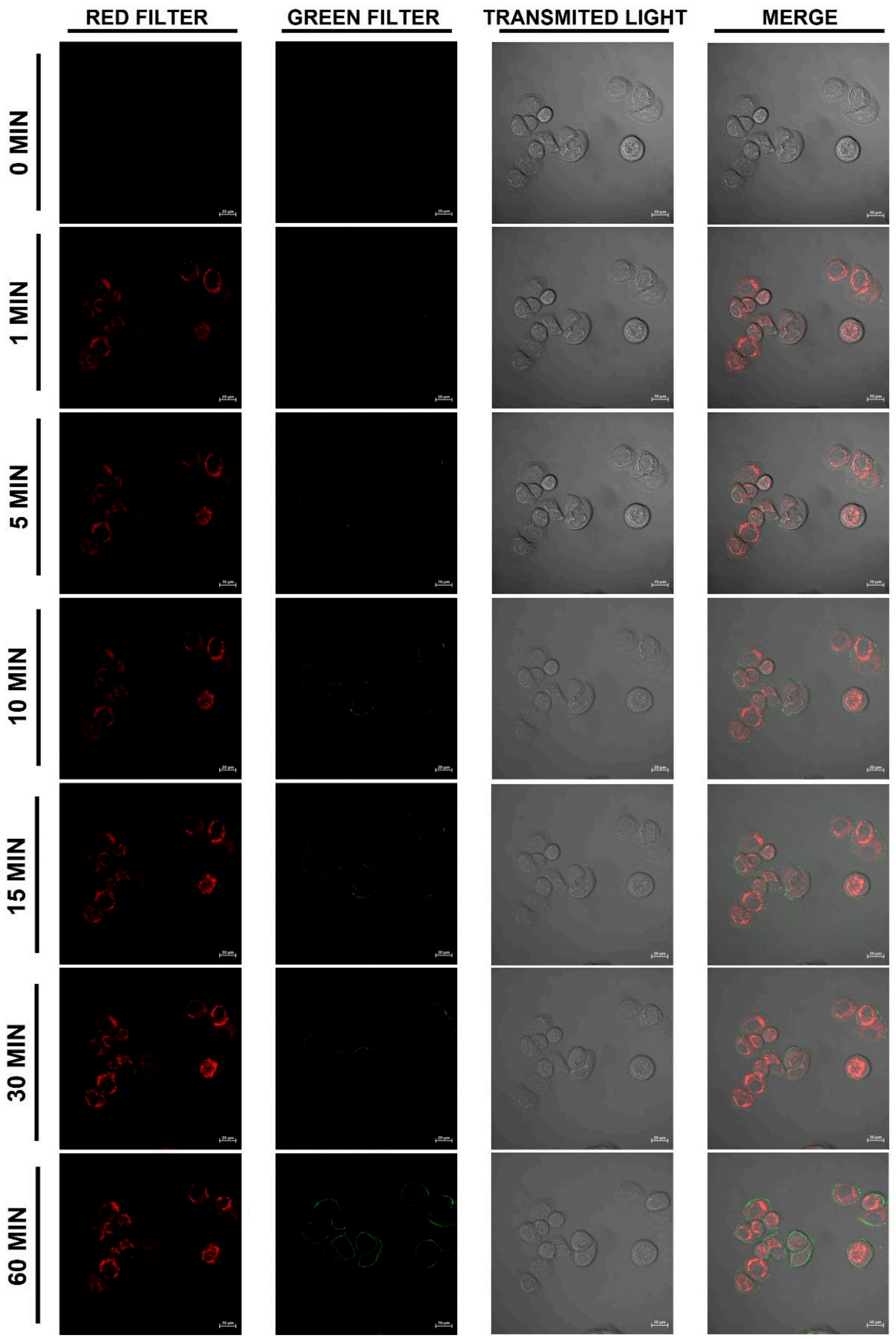


Figure S3. *Confocal microscopy of SKBR3 (scale bar = 20 µm) incubated with NR-LLNCs-HER2-PC. Bare LLNCs were used as control.*

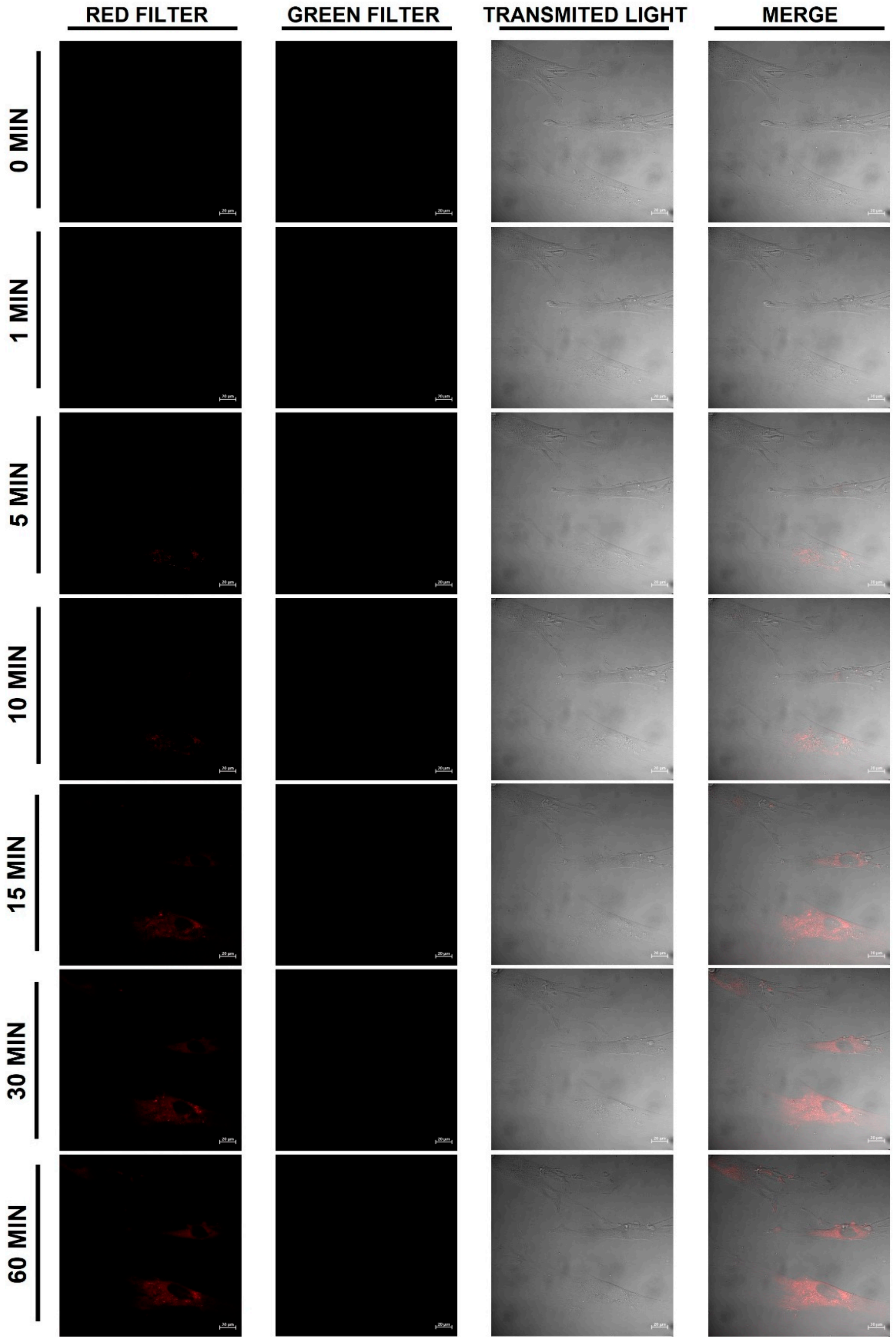


Figure S4. *Confocal microscopy of human Fibroblasts (scale bar = 20 μm) incubated with NR-LLNCs. Bare LLNCs were used as control.*

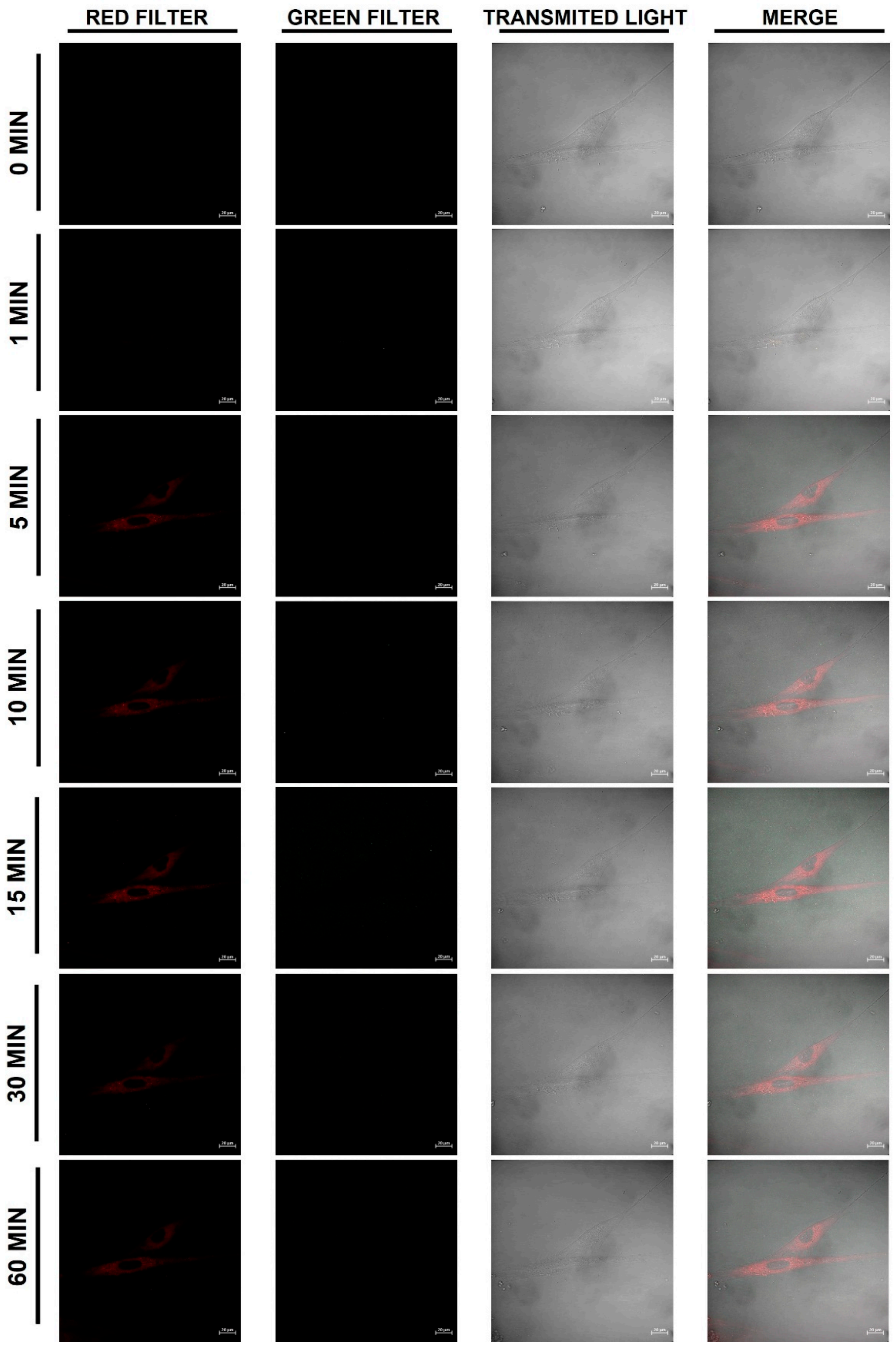


Figure S5. *Confocal microscopy of human Fibroblasts (scale bar = 20 µm) incubated with NR-LLNCs-HER2. Bare LLNCs were used as control.*

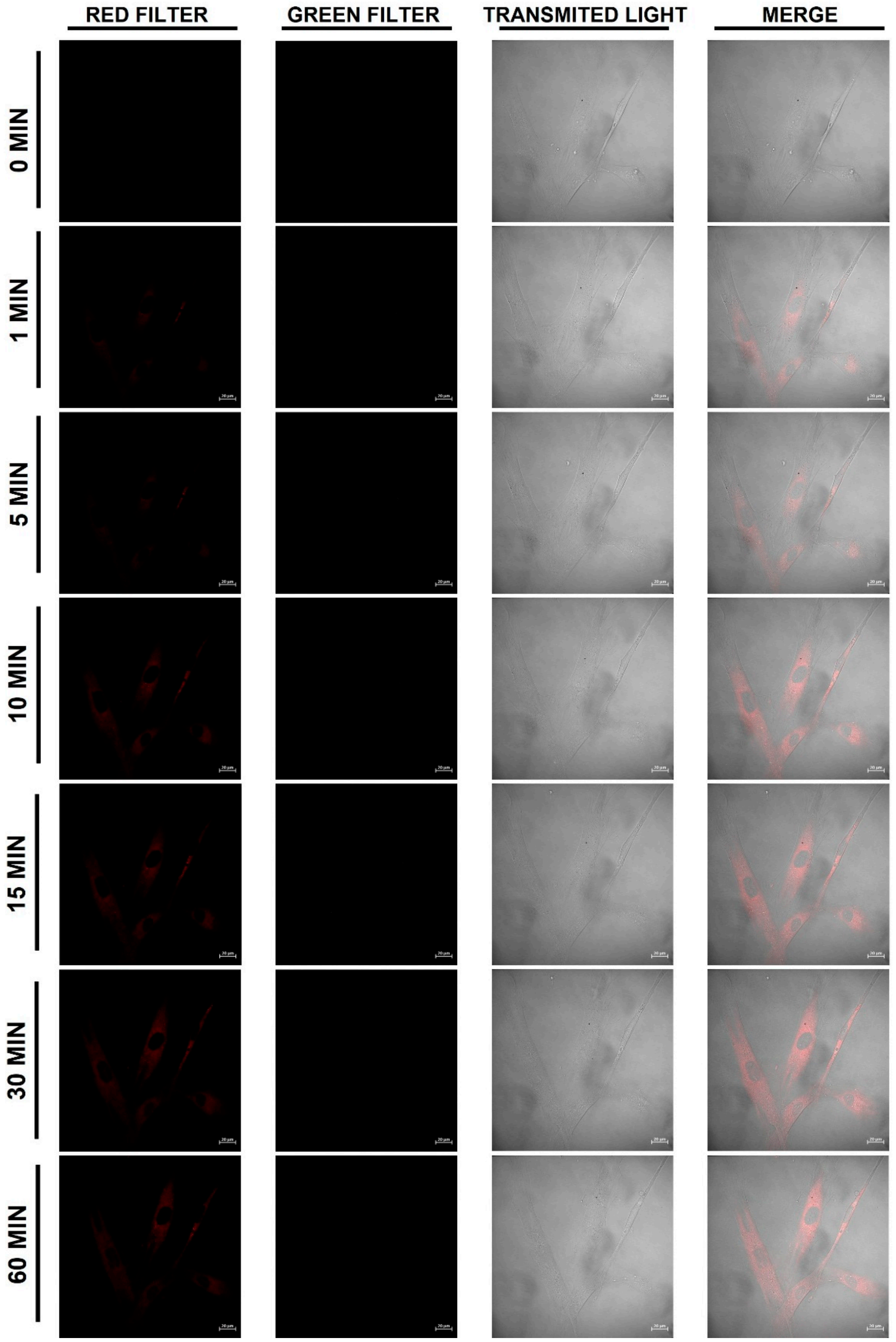


Figure S6. *Confocal microscopy of human Fibroblasts (scale bar = 20 µm) incubated with NR-LLNCs-HER2-PC. Bare LLNCs were used as control.*

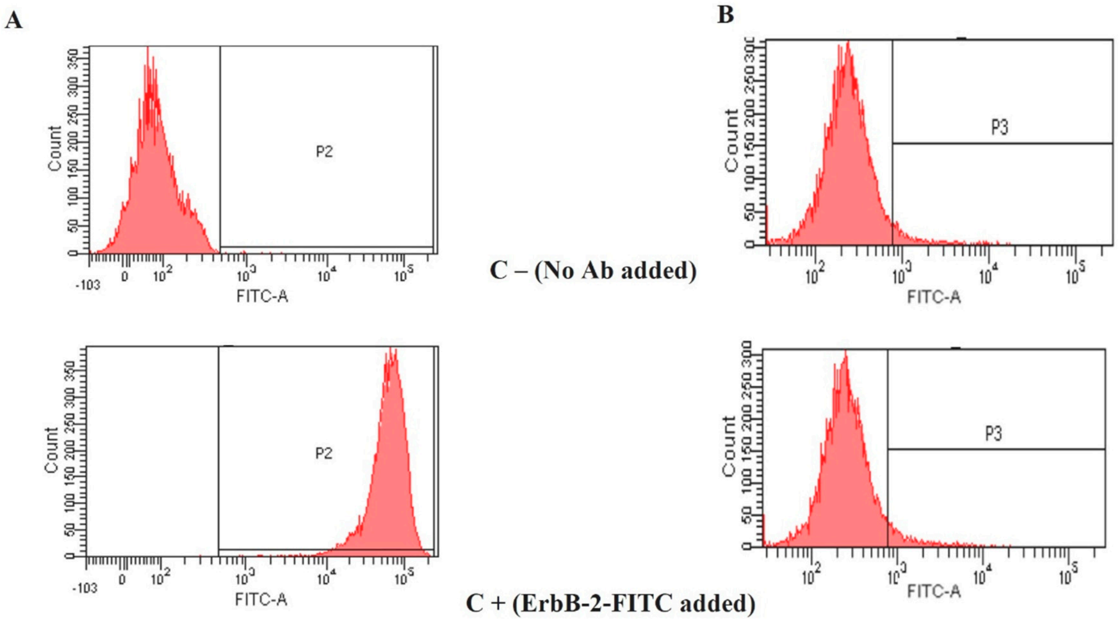


*Figure S7. Characterization of HER2 level expression in both SKBR3 and HDFa by flow cytometry. (A) Representative fluorescence intensity histograms of SKBR3. (B) Representative fluorescence intensity histograms of human HDFa.*

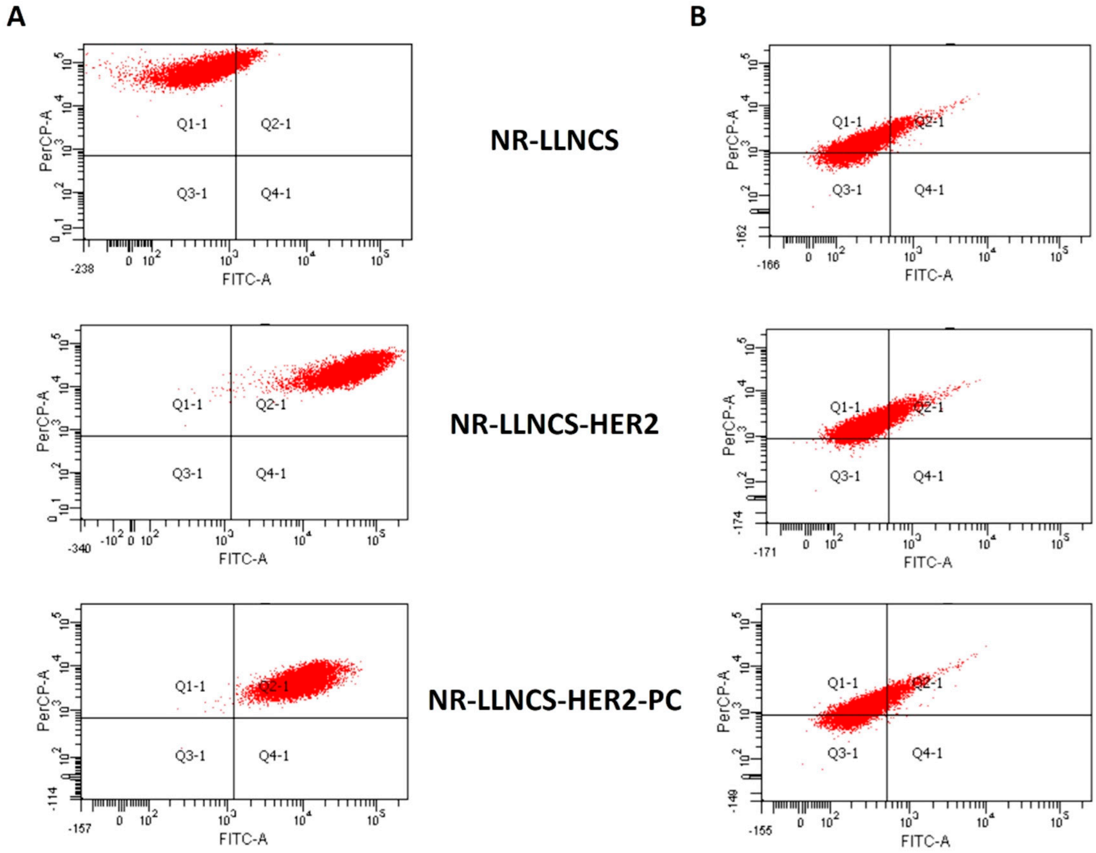


*Figure S8. Representatives dot plots of in vitro uptake of NR-LLNCs, NR-LLNCs-HER2 and NR-LLNCs-HER2-PC in both SKBR3 and HDFa at 60 mins by flow cytometry.* ***(A)*** *Representative dot plots of SKBR3.* ***(B)*** *Representative dot plots of human HDFa.*

A

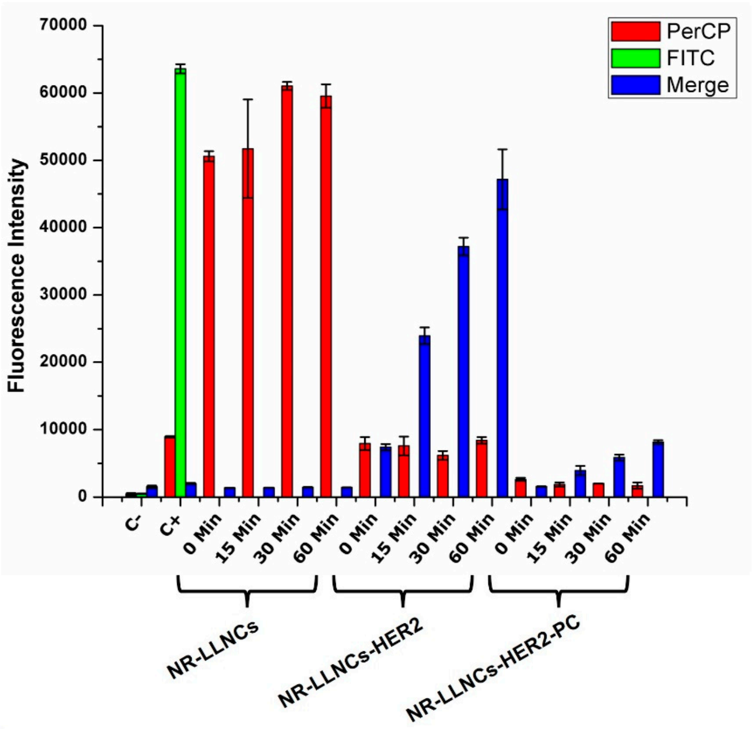


B

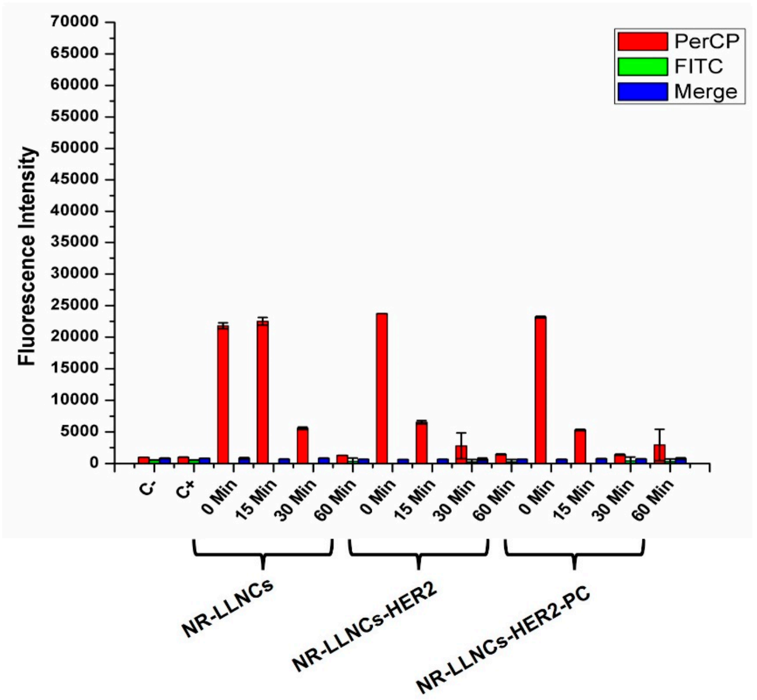


Figure S9. *Uptake of LLNCs, LLNCs-HER2 and LLNCs-HER2-PC in (A) SKBR3 and (B) HDFa analyzed by flow cytometry.*
